# Supplementary material for: Signal inference in financial stock return correlations through phase-ordering kinetics in the quenched regime
Source: PLoS One. 2025 Oct 31;20(10):e0334436. doi: 10.1371/journal.pone.0334436 (PMC12578216; doi:10.1371/journal.pone.0334436)
Supplement: Attachment — Submitted filename: Review_PONE_D_24_46297.pdf [file pone.0334436.s001.pdf]

Referee's report on the paper

PONE-D-24-46297 - Signal inference in financial stock return correlations through phase-ordering kinetics in the quenched regime

1 Introduction

This research provides a way to analyze financial data and potentially uncover hidden patterns in stock market behavior. These patterns could have important implications for investment strategies and risk management.

2 General comments

Although the merit of the work is recognizable, the way in which the results are presented to me does not seem very neat. Among the minor and easily correctable weaknesses are some spelling and grammatical errors found throughout the text, such as, for example:

1. P. 2: "ECM" is not defined.
2. Several pages. Some equations have boxes, while others do not. There are some equations that are not cited in the text and yet they are listed.
3. P. 5, eq. 23. The meaning of the symbol "$\approx$" should be explained.
4. P. 5, after eq. 28. The meaning of the symbol "$\sim$" should be explained.
5. Page 7: eq. 32. It does not correspond to an equation in its integral form.
6. Page 7, after eq. 32: "dW_t" is not a Wiener process.
7. Page 7, after eq. 33: it states: "we generate fully correlated walks using the same random seed value". Based on my knowledge, the use of a random seed ensures reproducibility of simulation results, but does not ensure that the generated trajectories are correlated, since the correlation structure is reflected in a variances and covariances matrix, for example.
8. P. 9: "continius".

Some major details that I found throughout the work are as follows.

1. The literature review presented in the Introduction is quite limited. If there are few papers in the area, this novelty should be highlighted. Regarding the use of Brownian motion in different models and scenarios, the literature is extensive.
2. The figures are disordered throughout the text. Before presenting each figure, there should be a brief introduction and then an explanation of the results obtained. However, the figures are found in the middle of paragraphs, and even on different sheets. This makes them difficult to understand.

Based on the review performed and the details presented, it is recommended to accept under major revisions.
