# Supplementary material for: Signal inference in financial stock return correlations through phase-ordering kinetics in the quenched regime
Source: PLoS One. 2025 Oct 31;20(10):e0334436. doi: 10.1371/journal.pone.0334436 (PMC12578216; doi:10.1371/journal.pone.0334436)
Supplement: Attachment — Submitted filename: Response to reviewers.pdf [file pone.0334436.s002.pdf]

Answer to referees comments

Date: 02 July 2025

Title: Signal inference in financial stock return correlations through phase-ordering kinetics

Author(s): Ixandra Achitouv, Vincent Lahoche, Dine Ousmane Samary

Report Id: PONE-D-24-46297R1 (v1)

Arxiv: 2409.19711

Dear referees, dear editor,

We thank the referees for their careful review and constructive comments. We detail below the changes made to the manuscript in response to their suggestions and hope they will appreciate this revised version. Please refer to the comments in blue, which highlight the main modifications.

Referee 1 We have carefully considered the referee comments and made significant revisions, which are highlighted in blue in the introduction. See the second paragraph of Introduction on the page 1 and 2

Referee 2 Regarding the comments from Referee 2, we found it quite challenging to summarize our previous results from reference [9] due to its length and technical depth. To remain focused on the main objectives of this manuscript, we have therefore provided a concise summary and omitted certain technical details that substantially overlap with that work.

1) We have modified the sentence following equation (15) to clarify that we provide a Wigner semi-circle distribution.

2) We have added a paragraph in Section VI to explain how the critical temperature can be computed, with reference to equations (30), (31), and (32).

3) We have also added a paragraph at the beginning of Section IV to clarify the main findings of our previous investigation concerning signal detection.

We would like to thank all the reviewers for their thoughtful comments, which have not only enabled us to thoroughly revise the manuscript, but have also greatly improved its overall quality.

On behalf of all the authors,

Dr Samary
